# Copan Walk Away Specimen Processor (WASP) Automated System for Pathogen Detection in Female Reproductive Tract Specimens

**DOI:** 10.3389/fcimb.2021.770367

**Published:** 2021-11-17

**Authors:** Jing Gao, Qiujing Chen, Yiqian Peng, Nanyan Jiang, Youhao Shi, Chunmei Ying

**Affiliations:** ^1^ Department of Clinical Laboratory, Obstetrics and Gynecology Hospital of Fudan University, Shanghai, China; ^2^ Institute of Cardiovascular Diseases, Ruijin Hospital, Shanghai Jiao Tong University School of Medicine, Shanghai, China

**Keywords:** Copan WASP, Copan-ESwab, female reproductive tract specimens, automation, diagnostic, bacteriology,

## Abstract

**Objective:**

Automation is increasingly being applied in clinical laboratories; however, preanalytical processing for microbiology tests and screening is still largely performed using manual methods owing to the complex procedures involved. To promote automation of clinical microbiology laboratories, it is important to assess the performance of automated systems for different specimen types separately. Therefore, the aim of this study was to explore the potential clinical application of the Copan Walk Away Specimen Processor (WASP) automated preanalytical microbiology processing system in the detection of pathogens in female reproductive tract specimens and its feasibility in optimizing diagnostic procedures.

**Methods:**

Female reproductive tract specimens collected from pregnant women at their first obstetric check-up were inoculated into culture media using the Copan WASP automated specimen processing system and were also cultured using a conventional manual inoculation method. After 48 h of culture, the growth of colonies was observed, and the types of bacteria, number of colonies, and efficiency in isolating single colonies were compared between the automated and manual groups. The specimens collected from the WASP system using the Copan-ESwab sample collection tubes were further analyzed for the presence of *Chlamydia trachomatis* (CT), *Neisseria gonorrhoeae* (NG), and *Ureaplasmaurealyticum* (UU) *via* fluorescence quantitative polymerase chain reaction (qPCR) and an immunochromatographic assay to investigate the feasibility of this method in optimizing detection of these common pathogens of the female reproductive tract.

**Results:**

Compared with the manual culture method, the Copan WASP microbiology automation system detected fewer bacterial types (*P*<0.001) and bacterial colonies (*P*<0.001) but had a higher detection rate of single colonies (*P*<0.001). There was no significant difference in the detection rates of common pathogens encountered in clinical obstetrics and gynecology, including group B *Streptococcus* (GBS) (*P*=0.575) and *Candida* (*P*=0.917), between the two methods. Specimens collected in the Copan-ESwab tubes could be used for screening of GBS and CT *via* fluorescence-based qPCR but not with immunochromatography. However, UU and NG were not detected in any sample with either method; thus, further validation is required to determine the feasibility of the Copan system for screening these pathogens.

**Conclusion:**

The Copan WASP microbiology automation system could facilitate the optimization of diagnostic procedures for detecting common pathogens of the female reproductive system, thereby reducing associated costs.

## 1 Introduction

Automation in clinical microbiology laboratories has long been attracting attention with rapid developments in recent years (Schubert and Kostrzewa, 2017). Currently, the identification and drug susceptibility tests for common pathogens are either fully automated or semi-automated (Singhal et al., 2015). The broad application of standardized operations has also facilitated the surveillance of drug resistance in bacteria and fungi (Pitout, 2018). However, the progress in achieving automation in processing preanalytical specimens has been relatively slow. The conventional manual method of specimen culture has apparent limitations in terms of systematic errors, process management, and personnel training and hence will inevitably be replaced by automation (van Belkum et al., 2013).

Currently, the two major manufacturers that dominate the global market of automated preanalytical microbiology processing systems are Copan Diagnostics, which developed the Walk Away Specimen Processor (WASP) system for specimen processing and inoculation, and BD Kiestra, which is a company branch devoted to building automated facilities for clinical microbiology laboratories (Croxatto et al., 2016). Their inoculation modules can be connected to laboratory information systems to enable the two-way communication of information systems and the standardization of preanalytical specimen processing procedures, thereby helping to reduce human errors during sample preparation and culture. In particular, procedures such as opening the lid of specimen containers, selecting appropriate culture plates based on the type of specimens, inoculating plates with the specimens (Iversen et al., 2016), closing the lid of specimen containers, and labeling for specimen classification can all be standardized *via* adjusting instrument settings to reduce labor-related costs, improve work efficiency, and reduce incubation times without affecting the test results (Cherkaoui et al., 2019). Hence, these modules represent an epoch-making change in the preanalytical processing of samples for microbial culture and identification (Croxatto et al., 2015). However, clinical microbiology tests are highly complicated processes involving diverse specimen types. Consequently, the customization of automated processing procedures for different types of specimens requires careful consideration and investigation by microbiologists (Croxatto et al., 2016).

To date, the application scope of automated microbiology specimen processing systems in China has covered various specimens, including blood, clean-catch urine, cerebrospinal fluid, sputum, nasopharyngeal swab (Tian et al., 2021), and feces; however, no study has reported the automated detection of pathogenic bacteria in female reproductive tract swab specimens. The female reproductive tract is an anatomical location characterized by a complex micro-ecological environment in which multiple microorganisms coexist (Al-Nasiry et al., 2020), thereby representing a research focus in the fields of microbiology, gynecology, and obstetrics. *Chlamydia* spp. and *Neisseria gonorrhoeae*, key pathogens of sexually transmitted disease (Graseck et al., 2011), are also the main causal pathogens of female pelvic inflammatory disease (Smolarczyk et al., 2021). Delayed diagnosis and treatment will eventually lead to an ectopic pregnancy and infertility (Gradison, 2012). Moreover, *Candida albicans* is an opportunistic fungal pathogen that colonizes the reproductive tract of 20% of women without causing any overt symptoms (Bradford and Ravel, 2017). *Candida* spp. are also the main pathogens of vulvo-vaginal candidiasis, with up to 75% of women becoming infected at least once in their lives (Willems et al., 2020). Group B *Streptococcus* (GBS) is a common pathogen responsible for infections of pregnant women and newborns and is closely related to preterm birth, stillbirth, and fetal injury (Armistead et al., 2019). Therefore, it is essential to accurately detect pathogens of the reproductive tract in a timely manner.

Our hospital attaches great importance to the development of new technologies and projects related to laboratory facilities; as part of this principle, our hospital recently acquired the first Copan WASP system in Shanghai for the inoculation and processing of female reproductive tract specimens. In this study, we compared pathogen detection results using the conventional manual method and the first-generation Copan WASP automated specimen processing system in 402 clinical obstetric and gynecologic samples collected for clinical microbiology tests. We also comprehensively evaluated and validated the procedures for detecting common pathogens in obstetrics and gynecology. Additionally, we explored the optimization of diagnostic procedures to determine whether the same specimen could be tested using various diagnostic methods at the same time to establish and promote automated microbial testing procedures (Fournier et al., 2013). Such standardization of automated pathogen detection could help to reduce unnecessary costs related to reagents, consumables, and labor, which can benefit both physicians and patients.

## 2 Materials and Methods

### 2.1 Source of Specimens

Reproductive tract specimens were collected from 402 pregnant women at 12–16 weeks of gestation during their first visits to the obstetric outpatient clinic of the Obstetrics and Gynecology Hospital of Fudan University (Shanghai, China) in March 2021.Women who had sexual intercourse in the last 72 h or those who were on an antibiotic treatment or who had a vaginal lavage in the 2 weeks prior to swab collection were excluded from the study.

The female lower genital tract secretions were collected using Copan-ESwab tubes, which is part of the Copan WASP automated preanalytical microbiology specimen processing system (Copan Italia S.p.a., Italy). Vaginal samples were obtained with vaginal swabs (Jiangsu Kangjian Medical Apparatus Co., Ltd.). The specimens were delivered to the laboratory within 0.5 h of collection and maintained at 25 ± 2°C for culture and testing.

The information of all samples could be identified during or after data collection. This study was performed in accordance with human subject protocols approved by the Ethics Committee of Obstetrics and Gynecology Hospital of Fudan University. All patients provided written informed consent to participate in this study.

### 2.2 Sample Inoculation and Culture

The specimens were inoculated into culture media using the Copan WASP automated specimen processing system *via* the 4 Quadrants-Type 3 continuous streaking mode. The specimens were also directly inoculated on culture plates manually by a senior clinical microbiologist in strict accordance with standard operating procedures. Blood agar (Comagal Microbial Technology Co., Ltd., Shanghai, China) culture plates for the isolation of common bacteria, including GBS and *Candida*, were incubated at 35°C in a common incubator, whereas *Neisseria gonorrhoeae* (NG) was cultured on selective medium (Yihua Medical Technology Co., Ltd., Shanghai, China) and incubated at 35°C with 5% carbon dioxide. *Ureaplasma urealyticum* (UU) was cultured using liquid medium and GBS was cultured on plates using the Copan WASP system.

### 2.3 Microbial Identification

After 48 h of culture, the manually and automatically inoculated samples were subjected to observation of colony morphology and colony counting. For specimens that yielded single colonies, each colony was directly isolated and subjected to matrix-assisted laser desorption/ionization time-of-flight mass spectrometry (MALDI-TOF MS; compass, Bruker Daltonics, Bremen, Germany). Specimens that did not yield single colonies were further subjected to purification and isolation to obtain single colonies, which were then subjected to mass spectrometry-based microbial identification the following day.

### 2.4 Screening for Common Pathogens in Female Reproductive Tract Specimens

GBS, *Chlamydia trachomatis* (CT), NG, and UU were detected *via* a fluorescence-based quantitative polymerase chain reaction (qPCR) assay, and the CT antigen was detected *via* an immunochromatographic assay. For qPCR, total nucleic acids were extracted from each sample using QIAamp DNA Mini Kit (Qiagen, NO.51304) according to the manufacturer’s instructions. PCR was performed in triplicate in 20 μl mixtures containing 2 μl of polymerase and 18 μl of reaction liquid (Jiangsu Bioperfectus Technologies Co. Ltd. and BioChain Co. Ltd.). PCRs were performed on an ABI 7500 Real-Time PCR System (Thermo Fisher Scientific, Waltham, MA, USA) using the following cycling parameters: 37°C for 5 min with uracil-DNA glycosylase, 95°C for 5 min, followed by 40 cycles at 95°C for 10s and 55°C for 40s (Sun et al., 2021). The products were analyzed on the Cobas z480 Analyzer (F. Hoffmann-La Roche Ltd., USA).

For the immunochromatographic assay, the anti-CT lipopolysaccharide monoclonal antibody and goat anti-rat IgG polyclonal antibody were fixed onto a nitrocellulose filter membrane for detection based on the double-antibody sandwich method, as reported previously (Naeem et al., 2021).

### 2.5 Statistical Analysis

The detection of pathogens in the female reproductive tract samples cultured using different methods were compared using a paired sample t-test for continuous variables or the chi-square test for count data (expressed as percentages) in SPSS 22.0 statistical software. *P*<0.05 indicated the presence of significant differences.

## 3 Results

### 3.1 Pathogen Detection

**Figure 1:**
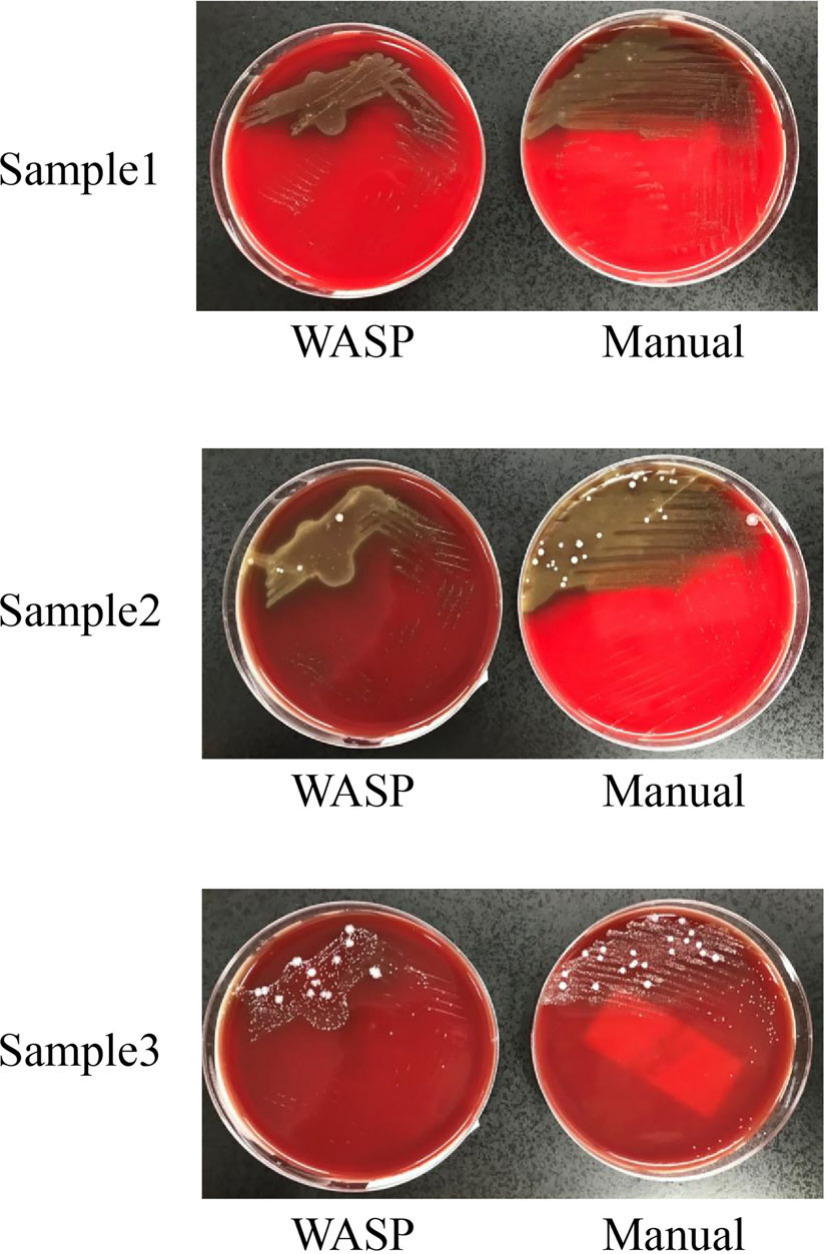
Difference in streaking patterns between Copan wasp and manual method.

**Figure 2:**
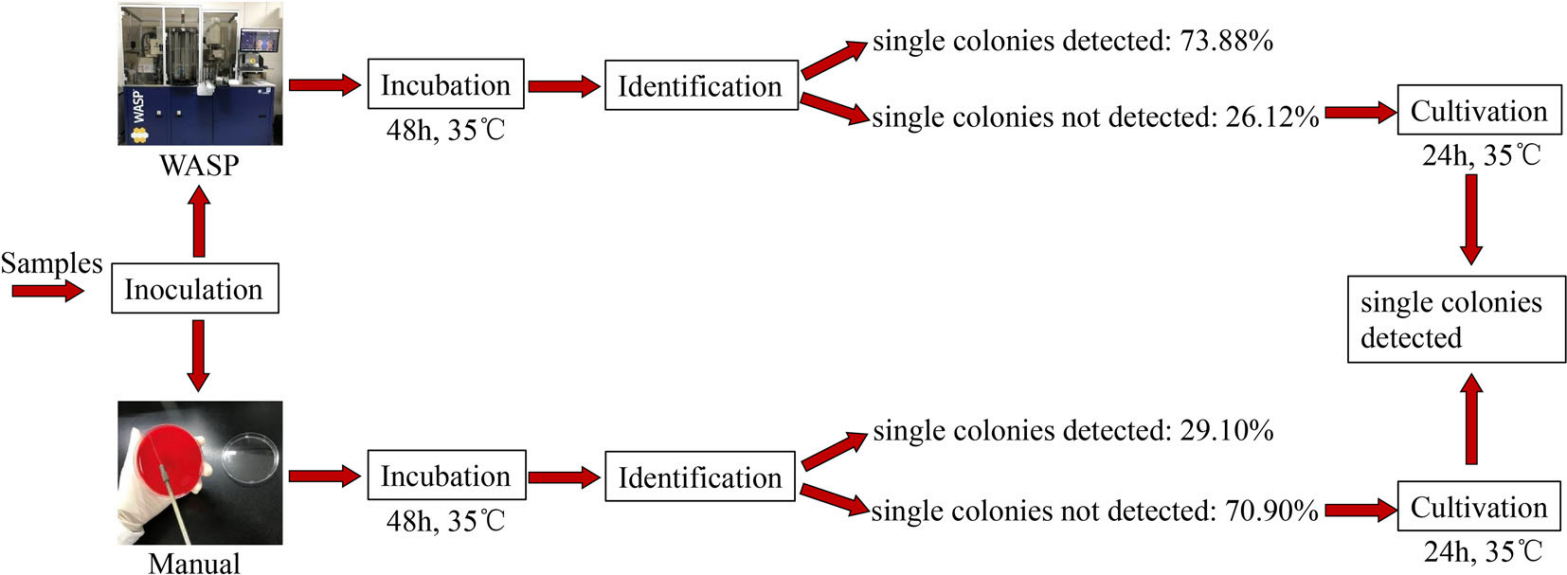
Difference in detection rate of single colonies between Copan wasp and manual method: For 402 samples we detected, 73.88% of them could directly incubate single colonies for identification within 48 hours by using Copan WASP, but this rate was only 29.10% if using manual method. It demonstrated that the identification result could be gained more quickly by using Copan WASP (*P*< 0.001).

Significantly more bacterial types were detected with the manual culture method than with the Copan WASP automated specimen processing system (2.25 ± 1.23 vs. 1.93 ± 1.08, *P*< 0.001; see **Table 1**). Significantly higher numbers of bacterial colonies were also detected with the manual method than with the Copan WASP system (422.77 ± 231.65 vs. 173.79 ± 126.25, *P*< 0.001; see **Table 1** and **Figure 1**). Both methods could detect *Candida*(especially *C. albicans*), GBS, *Lactobacillus* (especially *Lactobacillus crispatus*), *Staphylococcus*, Enterobacteriaceae (especially *Escherichia coli*), and *Enterococcus* (especially *Enterococcus faecalis*). However, *Corynebacterium* was readily detected by the manual method, with only few of these bacteria detected by the Copan WASP system. There was no difference in bacterial morphology *via* visual observation when the same specimens were inoculated on the plates using the two methods. However, the automated inoculation method had a significantly higher detection rate of single colonies (*P*<0.001) (**Table 1** and **Figure 2**).

**Table 1:** The types of bacteria, number of colonies and comparison of the detection rate of single colonies detected using manual and automated inoculation methods.

Female lower genital tract specimens (n=402)	Manual inoculation	Copan WASP	*P-value*
Types of bacteria detected per specimen (mean ± SD)	2.25 ± 1.23	1.93 ± 1.08	<0.001
Number of bacteria detected per specimen (mean ± SD)	422.77 ± 231.65	173.79 ± 126.25	<0.001
Detectable single colonies (n/%)	117/29.10	297/73.88	<0.001
Undetectable single colonies (n/%)	285/70.90	105/26.12	

### 3.2 Isolation and Culture Efficiencies of Common Pathogens

There was no significant difference in the detection rates of GBS (*P*=0.737) and *Candida* (*P*=0.917) between the manual and automated inoculation methods (**Table 2**). NG was not detected using either method; a negative coincidence rate of 100% was observed.

**Table 2:** Comparison of the isolation and cultivation efficiencies of common pathogens.

Pathogen species	Detection status	Manual inoculation	Copan WASP	*P-value*
GBS	positive(n/%)	20/4.98	17/4.23	0.737
	negative(n/%)	382/95.02	385/95.77	
Candida	positive(n/%)	54/13.43	53/13.18	0.917
	negative(n/%)	348/86.57	349/86.82	

### 3.3 Application of Copan-ESwab Tubes in Pathogen Detection *via* Fluorescence-Based qPCR

#### 3.3.1 Detection of CT

The female reproductive tract secretions collected using Copan-ESwab tubes were subjected to the detection of CT *via* a fluorescence-based qPCR assay and an immunochromatographic assay; the latter yielded a significantly lower detection rate than the former (*P* = 0.002). This suggested that such samples collected with Copan-ESwab tubes are not suitable for immunochromatographic detection but can be used for the nucleic acid detection of CT *via* fluorescence-based qPCR (**Table 3**).

**Table 3:** Application of Copan-ESwab tubes in the detection of common pathogens in female urogenital tracts *via* a fluorescence-based qPCR assay, immunochromatographic and culture.

Pathogen species	Inoculation method	qPCR	Immunochromatographic	Culture	*P-value*
CT	positive(n/%)	13/3.23	1/0.25	Not Detected	0.002
	negative(n/%)	389/96.77	401/99.75	Not Detected	
UU	positive(n/%)	160/39.80	NotDetected	192/47.76	0.027
	negative(n/%)	242/60.20	NotDetected	210/52.23	
GBS	positive(n/%)	14/3.48	NotDetected	17/4.23	0.715
	negative(n/%)	388/96.52	NotDetected	385/95.78	

#### 3.3.2 Detection of NG, UU, and GBS

NG was not detected using either the manual or automated methods; a negative coincidence rate of 100% was observed. The culture method yielded a significantly higher UU detection rate than that obtained in the qPCR assay (*P*=0.023). The culture method also resulted in a slightly higher GBS detection rate than the PCR assay, but the difference was not significant (*P*=0.715) (**Table 3**).

## 4 Discussion

In recent years, advances in information systems and data networking have played a vital role in the processing of clinical laboratory data. However, the development of automated and streamlined microbiology operations lags far behind other specialties in clinical laboratories, and clinical microbiology laboratory tests are still highly dependent on manual operations owing to their complicated procedures. The standardization of operating procedures in laboratory tests is not only the keystone to improve the service quality of clinical medicine but is also required for refined laboratory management. Therefore, there has been an increase in the popularity of streamlined microbiology operations in clinical laboratories at all tiers of healthcare institutions worldwide in recent years. As an orientation toward health insurance policies, the United States attempted to establish a new model of comprehensive clinical microbiology laboratory services in the systems of numerous hospitals (Sautter and Thomson, 2015), which could quickly reduce turnaround times (TATs) without increasing the costs involved. This standardized model further enabled the integrated use of technologies such as MALDI-TOF MS, next-generation sequencing, and nucleic acid amplification tests, thereby providing an accurate method of etiological diagnosis for infectious disease physicians and improving the overall quality of medical services (Sautter and Thomson, 2015).

In this study, we inoculated specimens using two different methods: the automated method using the Copan WASP system and the manual method. After culture, the manual inoculation method yielded more types of common bacteria and significantly higher numbers of bacterial colonies than the automated inoculation method, but the latter showed a significantly higher detection rate of single colonies than the former. Hence, the Copan WASP system could play a significant role in the subsequent isolation of single colonies for microbial identification and rapid drug susceptibility tests by reducing the time required for further purification and isolation by 24 h, on an average. Previous studies demonstrated that automated preanalytical microbiology processing systems such as Copan WASP and BD Kiestra can improve the detection and recovery rates of single colonies from clinical fluid specimens such as clean-catch urine (Froment et al., 2014; Quiblier et al., 2016). The WASP image analysis software also enables effective and rapid processing of specimens that yield negative results (Faron et al., 2020), and the WASP Lab automation system could improve the screening efficiency of group A *Streptococcus* in respiratory tract specimens (Van et al., 2019); these findings are consistent with the results of the current study.

Copan-ESwab tubes are more conducive to automation and have a greater fluid-absorption capacity than conventional transport media. Copan-ESwab tubes have been reported to exhibit a remarkable capacity to preserve the viability of various viruses and bacteria (including anaerobic bacteria) (Tyrrell et al., 2016; Nagy et al., 2018). Copan-ESwab tubes can be used as a short-term storage container for anaerobic bacteria (Demuyser et al., 2018) as well as for the transportation and storage of non-tuberculousmycobacteria and *Nocardia* at room temperature (20–25°C) and refrigeration temperature (2–8°C) (Gandhi et al., 2019). Furthermore, Copan-ESwab tubes have been proposed as excellent alternatives to other sampling tubes for collecting respiratory tract specimens for the diagnosis of coronavirus disease (Corman et al., 2020; Deiana et al., 2020), and the resulting detection rate could be improved by optimizing the reaction system (Jørgensen et al., 2021). Copan-ESwab tubes effectively preserve *Escherichia coli* and *Klebsiella pneumoniae* but lack the ability to preserve *Pseudomonas aeruginosa* (Tops et al., 2020). In this study, we found no significant differences in the detection rates of GBS and *Candida* between the automated and manual inoculation methods when cultivating pathogenic bacteria from female reproductive tract specimens collected using Copan-ESwab tubes. Hence, the Copan WASP system could be used for the cultivation of GBS and *Candida*. However, NG was not detected in any of the specimens using either of the methods, with a negative coincidence rate of 100%. Therefore, it will be necessary to increase the sample size to confirm the reliability of the test results.

In recent years, the development of molecular biology techniques has markedly improved the performance of clinical microbiology tests. Molecular biology techniques, including fluorescence-based qPCR assays, have been widely applied for the clinical detection of various pathogens. Therefore, it is also necessary to investigate how Copan-ESwab can be used with different detection methods without affecting the outcomes of bacterial cultivation to improve its utilization rate in the detection of pathogenic microorganisms. Accordingly, we explored the application of Copan-ESwab in the detection of common pathogens in the female reproductive tract *via* fluorescence-based qPCR.

GBS colonization or infection in the urogenital tract of women of childbearing age has remained a focus of research in obstetrics and gynecology. A multicenter study confirmed the presence of intermittent GBS colonization during pregnancy (Davies et al., 2004). After 24 h of culture at room temperature, the results of the BD-Max-GBS assay for the detection of GBS in samples collected using Copan-ESwab tubes were compared with those of the PCR assay following Lim broth enrichment and the conventional culture method. The comparison revealed no significant difference between the use of Copan-ESwab tubes and PCR following Lim broth enrichment (Silbert et al., 2016). The application of the WASP automated processor has significant advantages in the identification of GBS (Baker et al., 2020). We previously assessed the performance of Pheno Matrix digital imaging software in the detection of GBS from recto-vaginal swabs plated on a specific chromogenic medium using the WASP automated processor, demonstrating a sensitivity of 100% and specificity of 64.5% (Foschi et al., 2021). Our present results are consistent with the findings of these previous studies, wherein no significant difference was observed in the detection rate of GBS between the fluorescence-based qPCR assay and the culture method for samples collected using Copan-ESwab tubes. Hence, fluorescence-based qPCR can be used as an alternative to the conventional culture method to obtain qualitative results for samples collected using Copan-ESwab tubes with significantly shortened TATs, thus providing accurate and rapid reports in clinical practice.

Copan-ESwab has broad application potential with certain advantages in bacterial preservation. For instance, a previous study using Copan-ESwab for GBS detection of vaginal/rectal swabs on the WASP platform revealed that the sensitivity reached up to 93.8%, even increasing to 96.9% after enrichment (Buchan et al., 2014). *Escherichia coli* and *Enterococcus faecalis* exhibited resistance to repeated cycles of freezing (–80°C) and thawing in Copan-ESwab tubes, and fewer freeze-thaw cycles yielded better preservation of bacterial viability (Saliba et al., 2020). Copan-ESwab could also effectively maintain the viability of filamentous fungi for at least 48 h (Gandhi et al., 2018). In the present study, Copan-ESwab tubes preserved *Candida*, the main pathogen causing urogenital tract inflammation in women. Copan-ESwab also has notable application value in the screening of drug-resistant bacteria. For instance, Copan-ESwab could effectively screen carbapenemase-producing Enterobacteriaceae, especially KPC-producing *Klebsiella pneumoniae* (Foschi et al., 2020), and has been used for the screening of extended-spectrum beta-lactamase-producing Enterobacteriaceae in pediatric patients (Jewoola et al., 2020) as well as polymyxin-resistant Enterobacteriaceae (Girlich et al., 2019). The WASPLab automation system could also significantly shorten the time required for the identification of vancomycin-resistant *Enterococcus* (Cherkaoui et al., 2019), with markedly reduced reported TATs.

However, Copan-ESwab also presents a few limitations that must be addressed. First, the results obtained using Copan-ESwab were consistent with the testing results of our hospital with respect to the detection rate in the PCR-based screening of CT; however, specimens collected using Copan-ESwab tubes yielded unsatisfactory results with an extremely low detection rate in the immunochromatographic screening of CT. Therefore, Copan-ESwab can only be used for the screening of CT *via* fluorescence-based qPCR and cannot be applied for screening *via* immunochromatographic assays. Second, the application of Copan-ESwab posed issues in the detection of UU, as the subsequent culture resulted in a significantly higher detection rate than that obtained in the PCR assay. Moreover, our study only included pregnant women at their first obstetric visits; NG was not detected in any of the samples, and a negative coincidence rate of 100% was observed. Therefore, subsequent studies should focus on increasing the sample size to confirm the application value of Copan-ESwab in pathogen detection *via* different methods.

Here, we explored the application and process optimization of the Copan WASP system in the culture-based and fluorescence-based qPCR detection of common pathogens in female reproductive tract specimens. We conclude that the Copan WASP system enables the rapid isolation of single colonies from female reproductive tract specimens with a shorter TAT than possible with the manual method. Copan-ESwab has good application value in the culture-based and molecular detection of GBS, the culture-based detection of *Candida*, as well as the molecular detection of CT. Furthermore, Copan-ESwab enables the simultaneous detection of multiple pathogens, which can greatly help to reduce laboratory costs and further facilitate process optimization, thereby improving the efficiency of the sample processing system.

## 5 Conclusion

Application of the Copan WASP microbiology automation system for the detection of pathogens in female reproductive tract specimens could improve the detection rate of single colonies but yielded relatively fewer bacterial types and bacterial colonies. Furthermore, the automated method could be applied for the screening of GBS, *Candida*, and CT, thus facilitating the optimization of diagnostic procedures and reducing the costs involved.

## Data Availability Statement

The original contributions presented in the study are included in the article/supplementary material. Further inquiries can be directed to the corresponding author.

## Ethics Statement

The studies involving human participants were reviewed and approved by the Ethics Committee of Obstetrics and Gynecology Hospital of Fudan University. The patients/participants provided their written informed consent to participate in this study. Written informed consent was obtained from the individual(s) for the publication of any potentially identifiable images or data included in this article.

## Author Contributions

JG and QC wrote the manuscript and analyzed the data. YP, NJ, and YS provided the samples and collected the clinical data. CY designed and coordinated the study. All authors contributed to the article and approved the submitted version.

## Funding

Financial support was provided by the National Nature ScienceFoundation of China (grant number: 81873970).

## Conflict of Interest

The authors declare that the research was conducted in the absence of any commercial or financial relationships that could be construed as a potential conflict of interest.

## Publisher’s Note

All claims expressed in this article are solely those of the authors and do not necessarily represent those of their affiliated organizations, or those of the publisher, the editors and the reviewers. Any product that may be evaluated in this article, or claim that may be made by its manufacturer, is not guaranteed or endorsed by the publisher.
